# National Association of Dental Faculties

**Published:** 1889-10

**Authors:** 


The following officers were elected:

President, S. B. Barfield, Macon; 1st Vice-Pres., R. B. Adair, Gainesville; 2nd Vice-Pres., W. G. Browne, Atlanta; Rec. Sec., C. A. Rider, Gainesville; Cor. Sec., L. D. Carpenter, Atlanta; Treasurer, H. A. Lowrance, Athens.

Gainesville was chosen as the next place of meeting, the second Wednesday in July, 1890.

### National Association of Dental Faculties.

The sixth annual session of the National Association of Dental Faculties was held in the Town Hall, Saratoga Springs, N. Y., commencing Monday, August 5, 1889, at 10 A. M.

The Executive Committee reported that credentials had been received in accordance with the resolution adopted last year from the following colleges:

Chicago College of Dental Surgery, Truman W. Brophy.

Indiana Dental College, J. E Cravens.

State University of Iowa, Dental Department, A. O. Hunt.

New York College of Dentistry, Frank Abbott.

Boston Dental College, J. A. Follett.

Harvard University, Dental Department, T. H. Chandler.

Ohio College of Dental Surgery, H. A. Smith.

University of Pennsylvania, Dental Department, Jas. Truman.

Baltimore College of Dental Surgery, R. B. Winder.

Dental Department of Southern Medical College, L. D. Carpenter.

Vanderbilt University, Dental Department, W. H. Morgan.

University Dental College, J. S. Marshall.

Missouri Dental College, W. H. Eames.

Kansas City Dental College, J. D. Patterson.

Dental College of University of Michigan, J. Taft.

Subsequently credentials were received from Pennsylvania College of Dental Surgery, represented by C. N. Peirce; Harvard University, Dental Department, Thos. Fillebrown; and Louisville College of Dentistry, J. Lewis Howe.

Columbian University, Dental Department, represented by J.

Hall Lewis, and University of Maryland, F. J. S. Gorgas, were elected members of the association. The application for membership of Royal College of Dental Surgeons of Ontario was reported favorably, but the Executive Committee expressed a doubt as to the propriety of admitting it owing to the title of the association, which would seem to confine membership to colleges in the United States.

Applications from American College of Dental Surgeons, Chicago ; College of Dental Surgery of the University of Denver, and College of Dentistry, Department of Medicine, University of Minnesota, were laid over one year under the rules.

After a long discussion the association adopted by a vote of twelve to six a rule requiring attendance upon three full regular courses, in separate years, before examination for graduation. By a vote of eighteen to one the length of the regular courses was made "not less than five months each."

The time when the new rule shall go into effect was, on motion of Dr. Truman, fixed at the beginning of the session of 1891–92. It was also ordered, on motion of Dr. Patterson, that the resolutions requiring attendance on three terms be published in the announcements of the various colleges for the session of 1890–91.

A committee consisting of Drs. Truman, Taft, Cravens, Brophy, and Howe, was appointed to take into consideration the equalization of college fees. The committee subsequently reported a partial tabulation of fees, with a recommendation that the minimum fee be fixed at $100 a year. The report was laid over under the rules and the committee continued.

Drs. Cravens, Marshall, and Patterson were appointed a committee to codify the rules and report next year.

Dr. Fillebrown, from the committee appointed to consider the request of the Baltimore College of Dental Surgery with reference to the granting of the degree D.D.S. to a prominent practitioner without attendance upon lectures, reported in favor of declining the request. The report was accepted.

On motion of Dr. Gorgas, amended by Dr. Brophy, it was ordered that the colleges of this association print the list of their matriculates at the previous session, with the States or countries from which they come, in their annual announcement, with an asterisk (*) opposite the names of those not in attendance and a foot-note stating the fact.

On motion of Dr. Truman, it was ordered that colleges making application for membership be notified by the Secretary that it will be necessary for them to appear by representative before the Executive Committee.

Dr. Marshall offered the following, which was adopted:

*Resolved*, That all applications for membership reported upon favorably by the Executive Committee shall lie over one year before final action may be taken thereon.

Dr. Abbott offered a resolution requiring colleges of this association, desiring to confer the honorary degree, to submit the names of the persons so to be honored to this association for approval. Adopted.

The committee on text-books reported that the work recently published by Dr. Fillebrown had not been submitted to the committee for approval. The report was accepted.

The committee also reported that they had examined the work on "Orthodontia" compiled by Dr. S. H. Guilford, and recommended that it be adopted as a text-book. The report was accepted.

On motion of Dr. Truman, the work on "Dental Chemistry," by Dr. Clifford Mitchell, was accepted formally as a text-book.

The following resolutions were laid over under the rules:

Offered by Dr. Brophy:

*Resolved*, That graduates in medicine who have not had at least two years' practice in operative and prosthetic dentistry, shall be required to attend the lectures and engage in the practice-work in these departments during two annual sessions previous to admission to the examinations for the dental degree.

Offered by Dr. Patterson:

*Resolved*, That after the session of 1891–92 a diploma from a reputable medical college shall entitle its holder to enter the second course in dental colleges of this association, but shall not entitle him to an entrance into the senior class.

The following officers were elected for the ensuing year:

James Truman, President; L. D. Carpenter, Vice-President;

J. E. Cravens, Secretary; A. W. Harlan, Treasurer; Frank Abbott, J. Taft, and F. J. S. Gorgas, Executive Committee.

The following committees were appointed: *Ad interim* committee, Drs. T. W. Brophy, R. B. Winder, and J. A. Follett; Committee on Schools, Drs. H. A. Smith, J. D. Patterson, J. Lewis Howe, W. H. Morgan, W. H. Eames.

Adjourned to meet at the call of the Executive Committee.

